# Travel Burden and the Direct Medical Costs of Urologic Surgery

**DOI:** 10.36469/9825

**Published:** 2016-02-03

**Authors:** Daniel J. Olson, John L. Gore, Kenn B. Daratha, Kenneth P. Roberts

**Affiliations:** 1 University of Washington, Seattle, WA; 2 Washington State University, Spokane, WA

**Keywords:** quality of care, prostatectomy, transurethral resection of prostate, radical cystectomy, health care cost

## Abstract

**Background:** Increased surgical volume is associated with better patient outcomes and shorter lengths of hospitalization. As a consequence, traveling to receive care from a high volume provider may be associated with better outcomes. However, travel may also be associated with a decision by the healthcare provider to increase the length of stay due to a decreased ability to return to the primary hospital should complications arise. Thus, research is needed to understand the relationship between the distance a patient must travel and their outcomes following urologic surgery.

**Objective:** The purpose of this study was to determine whether the distance a patient travels to receive urologic surgery is associated with their length of hospital stay and direct medical hospitalization costs.

**Methods:** This was a retrospective observational cohort study of 12 106 patients over 50 years of age undergoing transurethral resection of the prostate (TURP), radical prostatectomy (RP) or radical cystectomy (RC) in Washington State hospitals between 2009 and 2013. Distance traveled was determined by calculating the linear distance between zip code centroids of patient residence and the hospital performing their procedure. Patients were sorted into four groups classified by distance traveled (≤5 miles, 6-20 miles, 21-50 miles and ≥51 miles) and cost calculated using a charges-to-reimbursement ratio for each hospital. Statistical significance was determined using a Kruskal-Wallis test.

**Results:** Patients traveling greater distances had significantly lower median medical costs compared with patients who lived closer to the hospitals where they underwent TURP and RP (TURP: ≤5 miles, $6243 and ≥51 miles, $5105, p≤0.001; RP: ≤5 miles, $12 407 and ≥51 miles, $11 882, p≤0.001), whereas there was no significant difference for patients undergoing RC (≤5 miles, $27 554 and ≥51 miles, $26 761, p=0.17). Likewise, patients traveling greater distances had significantly lower median lengths of hospitalization for TURP and RP (TURP: p≤0.001, RP: p≤0.001), while there was no difference for RC (p=0.50).

**Conclusions:** Patient travel burden does appear to play a role in cost and length of hospital stay for select urologic procedures with variable levels of morbidity and recovery time. Although these findings are statistically significant, the magnitude of the effect is small.

## BACKGROUND

Access to urologic care has been increasingly centralized to urban centers.[1] Two common prostate diseases requiring surgical treatment are benign prostatic hyperplasia (BPH) and prostate cancer, treated by transurethral resection of the prostate (TURP) and radical prostatectomy (RP), respectively. Both are highly prevalent: over 60% of men in their sixties exhibit symptoms of BPH,[2] and it is estimated that there was 220 800 new cases of prostate cancer for the United States in 2014.[3] Medical facilities and specialists for urologic surgical procedures are rarely located in rural communities, requiring patients in need of these procedures to travel to a regional medical hub, typically located in an urban area.

Increased hospital and surgeon procedure volume is associated with better patient outcomes and shorter lengths of hospitalization for RP and lower incidence of blood transfusion and other perioperative complications after TURP.[4-9] Thus, traveling to receive care from a high volume provider can be associated with better health outcomes. However, travel may also be associated with longer lengths of stay and a decreased ability to return to their primary hospital should complications arise. Thus, as providers are increasingly measured by the costs of the care they provide, efforts are needed to understand the relationship between the distance a patient must travel and their health systems outcomes, as defined by cost and length of hospitalization, following urologic surgery.

The purpose of this study was to understand the implications of distance traveled on the health systems outcomes of length of hospital stay and cost of service for patients undergoing urologic surgery. We selected TURP, RP and radical cystectomy (RC) to examine procedures that range in patient access to available providers (more providers offer TURP, fewer offer RC) and morbidity (lowest for TURP, highest for RC).[10] We evaluated whether the proximity to urologic surgical care is related to the length of their postoperative outcomes pursuant to national efforts to understand provider-specific health care value.

## METHODS

### Patient Population

We performed a retrospective observational cohort review of patients that underwent TURP, RP and RC in an inpatient setting in Washington State between 2009 and 2013. Data was retrieved from the Washington Comprehensive Hospital Abstract Reporting System (CHARS). CHARS provides de-identified patient discharge information including age, diagnosis and procedure codes, length of hospitalization, billing charges, patient admission source and the ZIP codes of the patient residence and hospital of treatment. Complete race/ethnicity data was not available in CHARS for all years included in the current study.

We identified, using the 9th edition of the International Classification of Diseases, 9th revision (ICD-9), surgical procedure codes for TURP (ICD-9 code 60.2), RP (ICD-9 code 60.5) and RC (ICD-9 code 57.7). We required patients undergoing RP to have a concurrent diagnosis of prostate cancer (ICD-9 185, 185.0) and patients undergoing RC to have a concurrent diagnosis of bladder cancer (ICD-9 188, 233.7, 236.7, 239.4). Our cohort was limited to patients who scheduled their procedure electively, defined as physician or clinic referrals and discharge to home. Patients traveling from outside Hospital Referral Regions (HRR)[11] 344, 437, 438, 438, 440, 441, 442, which comprise the state of Washington and parts of Oregon, Idaho and western Montana, were excluded. Patients under 50 years of age were also excluded to assemble a cohort of patients that represent the typical age and comorbidity distribution for these procedures.

An exemption determination application was reviewed by the Institutional Review Board at Washington State University and based on the de-identified nature of the dataset, the study was determined to be exempt from Institutional Review Board (IRB) review.

### Outcome Variables

We measured the length of patient hospitalization using the date of hospital admission and the date of discharge. Costs were estimated using charges data reported to CHARS and applying the cost to charge ratio calculated by the Healthcare Costs and Utilization Project (HCUP) for each hospital in Washington State, yielding an estimated cost.[12]

### Independent Variables

Distances traveled for treatment were estimated using the ZIP code of the patient residence and the ZIP code of the hospital where they received treatment. Travel distance was calculated as the linear distance from the centroid of these ZIP codes.[13] Patients were placed into four travel distance groups: those traveling ≤5 miles, 6-20 miles, 21-50 miles, and ≥51 miles.

### Covariates

Patient level covariates that were available in CHARS included age, gender, and payer status. Age was retained as a continuous variable as each the three study procedures have different age ranges. Payer status was categorized into a public payer group including Medicare, Medicaid and dual enrollees, and a private payer group including commercial insurance holders and self-pay patients. Patient comorbidities were established using ICD-9 codes according to Elixhauser, et al.[14]

### Statistical Analysis

Potential group differences were examined using Pearson Chi-Square analysis for categorical variables, independent samples t-tests for symmetrically distributed continuous variables, and Kruskal-Wallis tests for non-symmetrically distributed categorical variables. Statistical analysis was performed using SPSS Statistics software (IBM, Armonk, NY).

## RESULTS

There were substantial differences between patients undergoing TURP, RP and RC (Table 1). Patients undergoing TURP were significantly older than those undergoing RP or RC. The age of the TURP and RC populations was reflected in the primary payer distribution; Medicare was the predominate payer for TURP and RC patients. Travel burden was greatest for RC patients, who accessed the fewest number of Washington State hospitals. The travel burden was least for TURP patients, who accessed care at essentially all acute care hospitals in Washington State. Given the lower population density in Eastern Washington compared to Western Washington, we determined the distance traveled by patients in each region and found them to be the same (TURP) or less (RP and RC) for patients in Eastern Washington (Table 1).

**Table 1.** Characteristics of Hospitalized Patients Select Surgical Urologic Procedures Discharged 2009-2013 (N=12 106)

| | TURP (n=4346) | | Prostatectomy (n=7015) | | Cystectomy (n=745) | | P-value |
|---|---|---|---|---|---|---|---|
| **Demographic** | | | | | | | |
| Mean Age (SD) | 72 | (10) | 62 | (7) | 68 | (10) | <0.001 |
| Gender Male (Percent) | 4346 | (100) | 7015 | (100) | 620 | (83) | <0.001 |
| **Payer Mix** | | | | | | | |
| Medicare (Percent) | 2725 | (62.7) | 2222 | (31.7) | 417 | (56.0) | <0.001 |
| Medicaid (Percent) | 110 | (2.5) | 108 | (1.5) | 39 | (5.2) | |
| Private (Percent) | 950 | (21.9) | 2820 | (40.2) | 197 | (26.4) | |
| Other (Percent) | 561 | (12.9) | 1865 | (26.6) | 92 | (12.3) | |
| **Comorbidities** | | | | | | | |
| Hypertension (Percent) | 2101 | (48.4) | 2956 | (42.1) | 301 | (40.4) | <0.001 |
| Diabetes (Percent) | 837 | (19.3) | 755 | (10.8) | 86 | (11.5) | <0.001 |
| Chronic Lung (Percent) | 490 | (11.3) | 481 | (6.9) | 91 | (12.2) | <0.001 |
| **Distance Traveled (percent)** | | | | | | | |
| ≤5 miles | 1609 | (37.0) | 1516 | (21.6) | 134 | (18.0) | <0.001 |
| 6-20 miles | 1890 | (43.5) | 2517 | (35.9) | 200 | (26.8) | |
| 21-50 miles | 657 | (15.1) | 1812 | (25.8) | 260 | (34.9) | |
| 51+≤ miles | 190 | (4.4) | 1170 | (16.7) | 151 | (20.3) | |
| **Care Distribution** | | | | | | | |
| Number of Facilities | 53 | | 46 | | 28 | | |
| Treated Home HRR (Percent) | 3915 | (90.1) | 5520 | (78.7) | 534 | (71.7) | <0.001 |
| Median Travel Miles (IQR) | 9 | (4-17) | 16 | (6-34) | 24 | (9-42) | <0.001 |
| Median Travel Miles-wWA (IQR) | 9 | (4-15) | 17 | (7-33) | 24 | (10-40.5) | |
| Median Travel Miles-eWA (IQR) | 9 | (3-28.5) | 13 | (4-36) | 16 | (5-46.8) | |

HRR: Hospital Referral Region; IQR: Interquartile Range; TURP: transurethral resection of the prostate; wWA: Western WA; eWA: Eastern WA

Table 2 displays unadjusted costs of care and length of hospitalization stratified by travel burden. RC was associated with longer lengths of stay, a greater need for patients to travel out of their HRR for surgery, and higher costs, compared with TURP and RP. Although statistically significant differences for TURP and RP were observed in the analysis, these differences were small and negatively correlated (costs decreased) with distance traveled.

The unadjusted median estimated cost for all three procedures was found to be higher in Western Washington than in Eastern Washington. The unadjusted median estimated costs for TURP, RP and RC in Western Washington (HRRs 344, 437, 438, 439, and 441) were $6763 (IQR $5148 - $9390), $12 815 (IQR $10 020 - $16 408) and $29 980 (IQR $24 091-$40 646), respectively, compared with $4281 (IQR $3310 - $6067), $11 062 (IQR $8911-$15 924) and $22 491 (IQR $17 974-$34 960) in Eastern Washington (HRRs 440 and 442).

**Table 2.** Study Outcomes by Distance Traveled to Receive Select Surgical Urologic Procedures (N=12 106)

| Distance Traveled | | Hospitalization Length of Stay | | | Hospitalization Costs | | |
|---|---|---|---|---|---|---|---|
| **TURP (n=4,346)** | **n** | **Median** | **IQR** | **P-value** | **Median** | **IQR** | **P-value** |
| 0-5 Miles | 1609 | 1 | 1-2 | 0.007 | 6243 | 4495-8898 | <0.001 |
| 6-20 Miles | 1890 | 1 | 1-2 | | 6267 | 4633-8829 | |
| 21-50 Miles | 657 | 1 | 1-2 | | 6052 | 4479-8631 | |
| 50+ Miles | 190 | 1 | 1-2 | | 5105 | 3475-7258 | |
| **Prostatectomy (n=7,015)** | **n** | **Median** | **IQR** | **P-value** | **Median** | **IQR** | **P-value** |
| 0-5 Miles | 1516 | 2 | 1-2 | <0.001 | 12 407 | 9691-17 901 | <0.001 |
| 6-20 Miles | 2517 | 2 | 1-2 | | 12 930 | 9883-17 298 | |
| 21-50 Miles | 1812 | 1 | 1-2 | | 12 213 | 9746-15 096 | |
| 50+ Miles | 1170 | 1 | 1-2 | | 11 882 | 9369-14 548 | |
| **Cystectomy (n=745)** | **n** | **Median** | **IQR** | **P-value** | **Median** | **IQR** | **P-value** |
| 0-5 Miles | 139 | 9 | 7-12 | 0.50 | 27 554 | 21 413-39 699 | 0.17 |
| 6-20 Miles | 205 | 8 | 7-11 | | 29 073 | 21 981-40 681 | |
| 21-50 Miles | 268 | 8 | 7-12 | | 29 924 | 23 171-39 817 | |
| 50+ Miles | 165 | 8 | 7-10 | | 26 761 | 21 247-35 529 | |

IQR: interquartile ratio; TURP: transurethral resection of the prostate

## Discussion

The major finding of this study was that, in Washington State, the distance a patient traveled to undergo TURP and RP significantly affected their estimated care costs and length of hospitalization while it did not affect patients undergoing RC. We selected inpatient urologic procedures with a range of invasiveness and morbidity. TURP is minimally invasive and geographically the most widely available procedure in this study. RP is an intrapelvic procedure that is often performed laparoscopically with robot assistance and is only slightly less available than TURP. RC is the most complex of the studied surgeries, most often performed open, and has the highest morbidity of the three in large part due to the necessitated urinary diversion that accompanies removal of the bladder. RC is also the least geographically available of the three procedures considered, consistent with prior analyses demonstrating a natural regionalization of RC care.[15] Confounding factors found to influence postoperative outcomes after these procedures included patient age, preexisting comorbidities, and HRR.

Several studies have investigated postoperative complications and length of stay following TURP and RP.[5,16] Our study focuses on the effect of distance traveled and includes estimated costs as an important, measureable outcome. Previous studies demonstrated decisions for treatment at the closest or a more distant medical center are determined by a variety of factors: desire for treatment at centers with high surgeon and hospital volumes, option to avoid centers with perceived quality care differences, motivation for aggressive treatment, and travel distance considerations.[4,17,18] Our study compliments these by asserting that whatever the patients' motivation to travel further for their care, the distance they travel will not increase their hospital costs or length of stay.

The state of Washington is geographically diverse, with large contiguous urban centers in the West contrasted with large, rural areas dotted with a few urban centers in the East. It has large regional differences in the distribution of clinical care, especially in the surgical subspecialties. Thus, the distribution of care is an important consideration for residents of this state seeking access to surgical subspecialty procedures. Our results suggest that surgical subspecialty care, at least for urologic procedures, can be concentrated without negative consequences to patients based on the distance they travel for care.

There are reasons to believe that patients traveling greater distances would have higher costs and longer lengths of hospitalization including physicians being overly cautious and conservative in their treatment plans, ordering extra days in the hospital or unnecessary tests to reassure themselves that their patient is fit for discharge before their long trip home to areas where follow-up care might be scarce. Several studies have suggested that this is the case. It has been shown that patients traveling to the Mayo Clinic had worse outcomes when traveling a longer distance to receive care.[19] Likewise, two studies by Jackson et al. demonstrated an effect of distance traveled on length of stay for elective colorectal and pancreatic surgery.[20,21] These studies differed from ours in that the distances traveled in our study were much less (longest average distance 25 miles compared with an average distance over 200 miles).[19-21] Within the distances traveled in our study, a concentrated specialized care model is cost effective and does not negatively affect outcomes, although it may be inconvenient and incur increased personal costs for patients.

The small but significant negative correlation between cost and increased distance traveled for TURP and RP was unexpected. One possible explanation is that the costs for the three procedures used in this study were higher in Western Washington compared to Eastern Washington, and mixing patients from these two regions influenced the outcome. However, a separate analysis of the data on patients from Eastern Washington and Western Washington did not change the outcome (data not shown).

Our study had several limitations that should be considered when interpreting our findings. First, our study incorporated three urologic procedures performed in hospitals in the state of Washington and cannot be generalized for all surgeries performed in all states. We feel, however, that we have provided a model that could be applied in other states/regions and by which other procedures and geographic locale could be examined in a similar fashion. Other states have similar geographies with large urban areas and extensive rural regions. Second, the CHARS database only provided the charges for each case from which we used ratio of cost to charges, based on a set of assumptions, to calculate the estimated costs. However, our method for estimating costs is thorough in taking into account hospital diversity and is generalizable, providing us with conservative estimates of costs. Third, the CHARS database only reports patients that had surgery and were admitted to hospitals. Cases performed in ambulatory surgical centers were not included in our study. This, in addition to the increasing optimization of medical management of BPH, may partially explain the lower patient population in the TURP group. Also, the CHARS database does not provide surgeon volume, post-surgical complications or any other quality of care measures, which could have provided better insight into the relationships that we observed in our study. However, despite lacking these pieces of information, our study provides a strong framework upon which future studies can build, looking more closely at these measures from other data sources. Finally, the distances used in our analysis were also estimated as CHARS only provides a patients zip code in its database. By estimating linear distances we provided conservative estimates that did not take into account actual travel routes, which would have substantially increased the reported mileages.[13]

## CONCLUSION

This study demonstrates that the distance a patient travels to receive TURP and RP have a clinically significant relationship to lower medical costs or shorter length of hospitalization while RC does not. These results suggest that travel distance may not need to be considered when using administrative data to quantify quality or value in clinical services. Among the select number of patient level variables included in our study, the patients' age and HRR were found to play the greatest roles in increasing care costs and length of stay. Further research is needed to determine if these findings are unique to TURP, RP, and RC or if they can be generalized. Additional analysis is needed to better understand the major cost differences between the HRRs and hospitals in the state of Washington.

## CONFLICT OF INTEREST DECLARATION

The authors declare no conflict of interest.
